# Cannabichromene as a Novel Inhibitor of Th2 Cytokine and JAK/STAT Pathway Activation in Atopic Dermatitis Models

**DOI:** 10.3390/ijms252413539

**Published:** 2024-12-18

**Authors:** Ki Chan Kim, Ga Hee Jeong, Chul Hwan Bang, Ji Hyun Lee

**Affiliations:** 1 Department of Medical Sciences, Graduate School of The Catholic University of Korea, Seoul #222 Banpo-daero, Seocho-gu, Seoul 06591, Republic of Korea; kimkichan01@naver.com (K.C.K.); worldgh27@naver.com (G.H.J.); mrbangga@catholic.ac.kr (C.H.B.); 2 Department of Dermatology, Seoul St. Mary’s Hospital, College of Medicine, The Catholic University of Korea, Seoul #222 Banpo-daero, Seocho-gu, Seoul 06591, Republic of Korea

**Keywords:** atopic dermatitis, cannabichromene, phytocannabinoid

## Abstract

Cannabichromene (CBC) is one of the main cannabinoids found in the cannabis plant, and although less well known than tetrahydrocannabinol (THC) and cannabidiol (CBD), it is gaining attention for its potential therapeutic benefits. To date, CBC’s known mechanisms of action include anti-inflammatory, analgesic, antidepressant, antimicrobial, neuroprotective, and anti-acne effects through TRP channel activation and the inhibition of inflammatory pathways, suggesting that it may have therapeutic potential in the treatment of inflammatory skin diseases, such as atopic dermatitis (AD), but its exact mechanism of action remains unclear. Therefore, in this study, we investigated the effects of CBC on Th2 cytokines along with the Janus kinase (JAK)/signal transducer and activator of transcription (STAT) pathways involved in AD pathogenesis. We used a 2,4-Dinitrochlorobenzene (DNCB)-induced BALB/c mouse model to topically administer CBC (0.1 mg/kg or 1 mg/kg). The results showed that skin lesion severity, ear thickness, epithelial thickness of dorsal and ear skin, and mast cell infiltration were significantly reduced in the 0.1 mg/kg CBC-treated group compared with the DNCB-treated group (*p* < 0.001). In addition, real-time quantitative reverse transcription polymerase chain reaction (qRT-PCR) analysis showed a significant decrease in the mRNA expression of Th2 cytokines (*TSLP*, *IL-4*, *IL-13*) and inflammatory mediators (*IFN-γ*, *IL-1β*, *IL-6*, *IL-17*, *IL-18*, and *IL-33*) (*p* < 0.05). Western blot analysis also revealed a significant decrease in JAK1, JAK2, STAT1, STAT2, STAT3, and STAT6 protein expression (*p* < 0.05). These results suggest that CBC is a promising candidate for the treatment of AD and demonstrates the potential to alleviate AD symptoms by suppressing the Th2 immune response.

## 1. Introduction

Atopic dermatitis (AD) is a chronic inflammatory skin disease characterized by recurrent eczematous lesions, severe pruritus, and impaired skin barrier function. It primarily affects children but can persist into adulthood, affecting the quality of life due to its relapsing nature and severe symptoms. The global prevalence of AD has increased significantly in recent decades, with estimates suggesting that up to 20% of children and 10% of adults are affected by AD [1,2,3,4]. AD has complex and multifactorial characteristics including genetic, immunological, and environmental factors. The core of AD pathogenesis is abnormal immune responses, particularly the dysregulation of T-helper cells. Overproduction of inflammatory cytokines, such as interleukin-4 (IL-4), IL-13, and IL-31, particularly Th2 cells, is a characteristic of AD, and overproduction of cytokines is known to activate the Janus kinase (JAK)/signal transducer and activator of transcription (STAT) pathway, which is the core of AD pathogenesis [5,6].

Therefore, targeted therapies, such as JAK inhibitors and biologics, that block specific cytokines involved in the inflammatory process have been developed to understand the molecular mechanisms of AD. Although these therapies offer promising results, their long-term efficacy and safety profiles remain under investigation. Furthermore, existing treatments such as corticosteroids and calcineurin inhibitors are often associated with side effects, leading to a continued search for safer and more effective alternatives [7,8,9].

Therefore, we sought to determine whether cannabichromene (CBC), a phytocannabinoid found in cannabis, affects AD. Although CBC is less well known than tetrahydrocannabinol (THC) and cannabidiol (CBD), the most widely recognized phytocannabinoids, it has recently attracted attention for its potential therapeutic effects. CBC is known to exhibit anti-inflammatory, analgesic, antidepressant, antimicrobial, neuroprotective, and anti-acne properties; however, the underlying mechanisms remain unclear [10,11,12,13].

**Figure 1:**
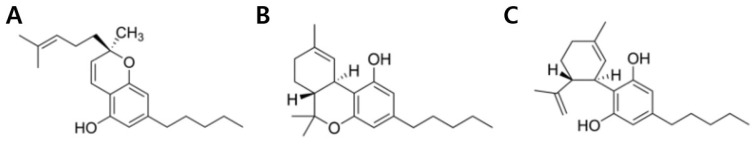
Chemical structures of (**A**) cannabichromene, (**B**) tetrahydrocannabinol and (**C**) cannabidiol.

As shown in Figure 1, CBC, THC, and CBD belong to the cannabinoid family and share a common chemical framework with subtle structural differences. These differences in chemical structure have a significant impact on the pharmacological properties and potency of each compound, which in turn determine the effects and side effects of each compound [14,15].

The first difference is that CBC and CBD have non-psychoactive effects, whereas THC causes psychoactive effects. THC’s primary effect is analgesia, whereas CBC and CBD are known for their anti-inflammatory properties. In terms of side effects, THC can cause mental side effects such as memory impairment, anxiety, and cognitive decline. CBD, on the other hand, has been reported to have mild side effects, such as drowsiness and diarrhea. CBC is still in the early stages of research; therefore, no specific side effects have been identified, but it has been suggested that it may cause fatigue or digestive discomfort in rare cases [16,17,18,19].

Although CBC is a relatively minor phytocannabinoid compared to THC and CBD, there are a lack of experimental data directly addressing its effects on dermatological conditions. Moreover, THC’s strong psychoactive effects limit its clinical applicability in real patients. In contrast, CBD has been extensively studied in patients with skin conditions. For instance, a study involving 20 patients with various skin conditions, including psoriasis (n = 5), AD (n = 5), and skin scarring (n = 10), demonstrated significant improvements in severity scores and skin moisture levels after CBD treatment [20]. Additionally, a large, double-blind, randomized, placebo-controlled trial in 51 psoriasis patients, though not specific to AD, found that those treated with 2.5% CBD ointment for 12 weeks experienced significantly lower psoriasis severity scores than the placebo group.

However, the effects of CBC have primarily been studied in vitro or in limited inflammatory models, leaving a gap in understanding its mechanism of action and therapeutic potential in skin diseases such as AD. To address this, we conducted a study using an AD mouse model to explore the potential effects of CBC on AD [21].

Our experiments were designed to test the hypothesis that CBC can modulate key inflammatory pathways, such as the JAK/STAT pathway, which plays a central role in the pathogenesis of AD. Through these studies, we aim to provide insights into CBC’s therapeutic potential and its mechanisms of action in inflammatory skin conditions like AD.

## 2. Results

### 2.1. Effect of Cannabichromene in AD Mouse Model

**Figure 2:**
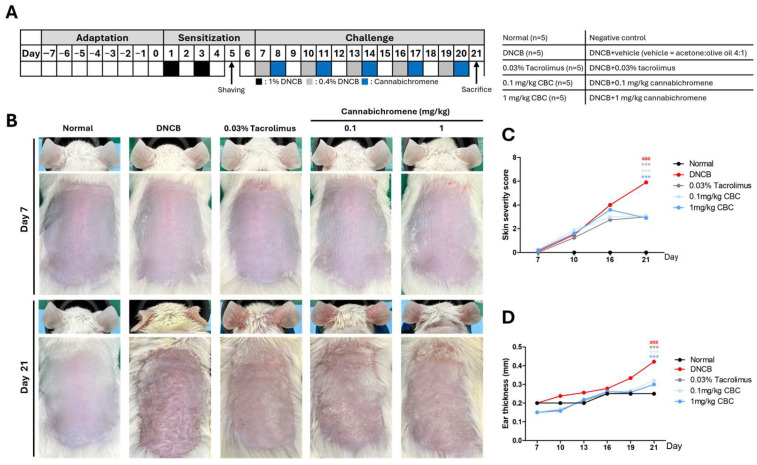
Atopic dermatitis mouse model experiment schedule and clinical experiment results. (**A**) Animal experiment schedule and group details, (**B**) Clinical photo on day 7 before the start of the challenge and on day 21 before the sacrifice, (**C**) Average score of 4 items: erythema, dryness, excoriation, and edema, (**D**) Average value of both ear thicknesses measured with a Dial thickness Gauge during the challenge period. The experiment was conducted in 5 groups, and a total of 25 animals were used, n = 5 per group. Data shown in the figure are presented as mean ± SEM (### *p* < 0.001 vs. normal group; *** *p* < 0.001 vs. DNCB group). DNCB, 1-chloro-2,4-Dinitrochlorobenzene.

After 7 days of adaptation, we performed sensitization by treating BALB/C mice twice with 1% 1-chloro-2, 4-dinitrobenzene (DNCB) on the abdomen. After that, we removed the hair from the back area to induce lesions of 2 × 2 size and treated the back and ears with 0.4% DNCB at 3-day intervals to create an AD mouse model. The day after treating the AD mouse model with DNCB, we treated the backs and ears with 0.03% tacrolimus and 0.1 mg/kg or 1 mg/kg CBC as a positive control. The effect of CBC in the AD mouse model was confirmed by comparing the group treated with DNCB alone with the group treated with 0.03% tacrolimus (Figure 2A). CBC was treated a total of five times during the 21-day experiment; when comparing the lesions of mice on day 7, the start day of the challenge, and day 21, the day of sacrifice, all concentrations of CBC-treated groups showed a significant decrease compared to DNCB (*p* < 0.001) (Figure 2B,C). In addition, when ear thickness was measured before DNCB treatment during the challenge period, all concentrations from the CBC-treated groups showed a significant decrease compared with DNCB (*p* < 0.001) (Figure 2B,D).

### 2.2. Inhibitory Effect of Cannabichromene on Epidermal Hyperplasia and Mast Cell Infiltration in AD Mouse Models

After the animal experiment, we prepared paraffin blocks of dorsal tissues and confirmed epidermal hyperplasia and inflammatory cell infiltration through hematoxylin and eosin (H&E) staining; we also performed pathological observations such as mast cell infiltration through staining with toluidine blue.

**Figure 3:**
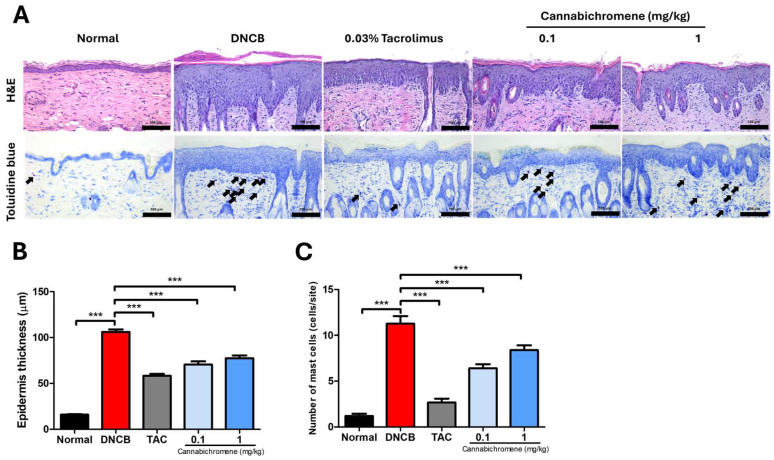
Result of staining after treating cannabichromene on atopic dermatitis mouse model dorsal skin. (**A**) Representative H&E stain and Toluidine blue stain images from each group (200× magnification, arrows indicate mast cells), (**B**) results of measuring epidermis thickness after H&E stain (total n = 5/group), (**C**) results of counting mast cell invasion (total n = 5/group), data shown in the figure are provided as mean ± SEM (*** *p* < 0.001 vs. normal group or DNCB group). DNCB, 1-chloro-2,4-Dinitrochlorobenzene; TAC, 0.03% tacrolimus; H&E, Hematoxylin and Eosin.

H&E staining confirmed that the epidermis was significantly thicker in the group treated with DNCB alone than in the untreated normal group (*p* < 0.001) (Figure 3A,B). The significantly thickened epidermis was significantly reduced in the 0.03% tacrolimus (*p* < 0.001), 0.1 mg/kg CBC (*p* < 0.001), and 1 mg/kg CBC (*p* < 0.001) groups compared to the DNCB group (Figure 3A,B), and toluidine blue staining results showed that the DNCB group had a lot of mast cell infiltration compared to the normal group (*p* < 0.001) (Figure 3A,C). Additionally, the infiltrated mast cell infiltration was significantly reduced in the 0.03% tacrolimus (*p* < 0.001), 0.1 mg/kg CBC (*p* < 0.001), and 1 mg/kg CBC (*p* < 0.001) groups compared to the DNCB group (Figure 3A,C).

These pathological experimental results showed that all concentrations of CBC showed significant effects, but the effect was better at a low concentration of 0.1 mg/kg than at 1 mg/kg.

### 2.3. Cannabichromene Showed the Effect of Reducing Cytokines Associated with AD in the AD Mouse Model

**Figure 4:**
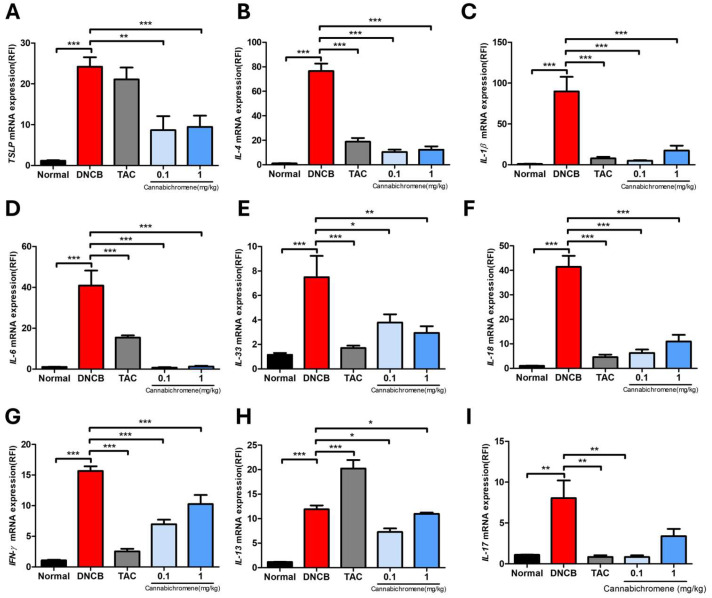
Results of confirming the mRNA expression of various cytokines after treating cannabichromene in an atopic dermatitis mouse model. Various cytokines (**A**) *TSLP*, (**B**) *IL-4*, (**C**) *IL-1β*, (**D**) *IL-6*, (**E**) *IL-33*, (**F**) *IL-18*, (**G**) *IFN-γ*, (**H**) *IL-13*, (**I**) *IL-17* were identified through qRT-PCR (total n = 5/group). All cytokines were normalized to *Actb*, and expression levels were analyzed using the ΔΔct method. Data shown in the figure are presented as mean ± SEM (* *p* < 0.05, ** *p* < 0.01, *** *p* < 0.001 vs. normal group or DNCB group). DNCB, 1-chloro-2,4-Dinitrochlorobenzene; TAC, 0.03% tacrolimus.

We performed quantitative real-time PCR (qRT-PCR) to confirm the expression level of mRNA in the dorsal tissue of mice sacrificed after the animal experiment and confirmed that the mRNA expression of all targets confirmed through the experiment was significantly increased in the DNCB group compared to the normal group. In addition, we confirmed that the remaining cytokines, *TSLP*, Th2-related *IL-4*, *IL-13*, inflammation-related *IL-1β*, *IL-6*, *IL-33*, *IL-18*, and *IFN-γ* (Figure 4A–H), significantly decreased, except for *IL-17*, in the 1 mg/kg CBC treatment group (*p* < 0.05) (Figure 4F). However, in the 0.1 mg/kg CBC treatment group, all targets, including IL-17, significantly decreased compared to the DNCB group. In particular, we confirmed that the 0.1 mg/kg CBC group was effective despite a lower concentration for most targets than the 1 mg/kg CBC group.

### 2.4. Effect of Cannachromene on JAK/STAT Pathway in AD Mouse Model

**Figure 5:**
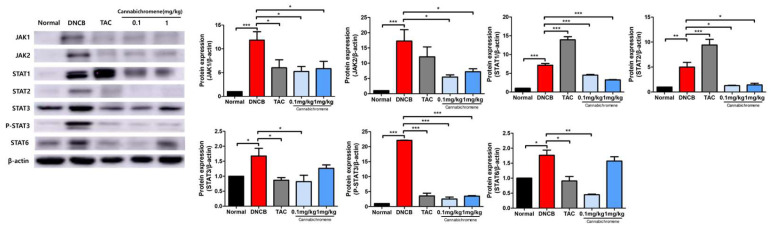
Results of confirming the expression of proteins related to the JAK/STAT pathway after treating cannabichromene in an atopic dermatitis mouse model. Through Western blot, the expression of JAK/STAT family of proteins (total n = 5/group) was confirmed. The membrane band intensity of all proteins was measured using ImageJ software and normalized to β-actin. Data shown in the figure are presented as mean ± SEM (* *p* < 0.05, ** *p* < 0.01, *** *p* < 0.001 vs. normal group or DNCB group). DNCB, 1-chloro-2,4-Dinitrochlorobenzene; TAC, 0.03% tacrolimus.

We also confirmed the effect of CBC on the JAK/STAT pathway, the most important contributor to the development of AD, by Western blot. The results showed that the levels of JAK1, JAK2, STAT1, STAT2, STAT3, Phospho-STAT3, and STAT6 were significantly increased in the DNCB-alone treatment group compared to normal, and the levels of JAK1, JAK2, STAT1, STAT2, STAT3, Phospho-STAT3, and STAT6 were significantly increased in the 0.1 mg/kg CBC treatment group significantly decreased the levels of JAK1, JAK2, STAT1, STAT2, STAT3, Phos-pho-STAT3, and STAT6 compared to the DNCB group (*p* < 0.05) (Figure 5).

These Western blot results demonstrate that CBC topical treatment inhibits JAK/STAT signaling, which is known to be of paramount importance in AD.

## 3. Discussion

The mouse model of DNCB-induced AD used in this study is commonly used in many studies on AD [22,23,24]. In our study, DNCB-treated mice showed increased skin severity scores and ear thickness compared to the normal group; histologically, they showed significantly increased epidermis thickness and mast cells. In addition, Th2 cytokines (IL-4 and IL-13) and JAK/STAT proteins, which are most representative of the development of AD, were significantly increased, suggesting that the mouse model used in our study is a suitable model for studying AD [8,25].

Tacrolimus, used as a positive control in this study, is a calcineurin inhibitor that does not directly target the JAK/STAT pathway but acts as an immunosuppressant by inhibiting T cell activation. However, previous studies have reported that tacrolimus can indirectly regulate the JAK/STAT pathway in certain situations. For example, it was shown to effectively inhibit Receptor activator of nuclear factor-κB ligand (RANKL) expression by inhibiting the JAK2/STAT3 pathway and upregulating SOCS3 in rheumatoid arthritis fibroblast-like synoviocytes (FLS) stimulated with IL-6/sIL-6R [26]. In addition, it inhibited IFN-α-induced nuclear translocation of STAT in hepatocytes [27]. Owing to these properties, tacrolimus is widely used to study inflammatory responses and immune regulation mechanisms in various animal models or to evaluate therapeutic effects. In this study, the JAK/STAT pathway inhibition effect was confirmed in the tacrolimus-treated group, which demonstrated that tacrolimus is a suitable positive control for AD research.

The cannabichromene (CBC) used in this study is one of the various extracts from the *Cannabis sativa* L. plant, and is a lesser-known phytocannabinoid than tetrahydrocannabinol (THC) and cannabidiol (CBD), which are the most well known, but are known to have anti-inflammatory effects [12]. CBC reduced interferon-γ (IFN-γ) and IL-10 expression when administered at 1 μm in macrophages induced by LPS, and subsequent intraperitoneal (I.P.) injection of 0.1 and 1 mg/kg of CBC into a DNBS-induced inflammatory disease (IBD) mouse model resulted in a potent reduction in inflammation [28]. The treatment of LPS-induced RAW 264.7 cells with CBC at concentrations of 5, 10, and 20 μm decreased iNOS, COX-2, IL-1β, IL-6, and TNF-α mRNA expression, respectively, and subsequent intraperitoneal (I.P.) injection of 1 and 10 mg/kg CBC in a mouse model of acute inflammation induced by λ-carrageenan confirmed the therapeutic effect of anti-inflammatory effects at these concentrations [29]. Finally, in a mouse model of acute respiratory distress syndrome (ARDS) induced by poly (I:C) treatment, 5 mg/kg CBC was administered with an inhaler, and the therapeutic effect was confirmed by decreasing the expression of IL-6, IL-17, and IFN-γ [30]. We confirmed that CBC exhibited potent anti-inflammatory effects from low doses of 0.1 mg/kg to high doses of 10 mg/kg in mouse models of various diseases; based on this, we set local doses of 0.1 mg/kg and 1 mg/kg and designed the study to evaluate whether the anti-inflammatory effect of CBC is effective in AD, a chronic inflammatory skin disease.

AD is characterized by skin thickening due to various stimuli, and it is known that mast cell activation by IgE and Th2 cytokines plays an important role in the inflammatory process [9,31]. In our experiments, treatment of thickened epidermis with two concentrations of 0.1 and 1 mg/kg CBC restored it to normal thickness and reduced the increased number of mast cells. These results suggest that CBC is an effective treatment for AD.

As previously described, the DNCB-induced AD mouse model is a common model used by many researchers to study AD. In our study, we found that the mRNA expressions of *TSLP*, *IL-4*, *IL-1β*, *IL-6*, *IL-33*, *IL-18*, *IFN-γ*, *IL-13*, and *IL-17* significantly increased in the DNCB-alone group and significantly decreased in the CBC group. TSLP, IL-4, and IL-13 are the major cytokines that activate Th2 immune responses, induce IgE production, and amplify the activity of mast cells and eosinophils, causing itching and inflammation [32,33,34]. IL-33 is mainly expressed in keratinocytes and skin immune cells and plays an important role in amplifying Th2 immune responses [35,36,37]. IL-1β and IL-6 are important cytokines in the early stages of inflammatory responses, stimulating keratinocytes and immune cells in damaged skin barriers and inflamed sites to induce a chain reaction of inflammatory cytokines [38,39]. IL-18 is expressed in damaged skin together with IL-33, regulating innate and adaptive immune responses, and is involved in inducing Th1 immune responses as well as Th2 immune responses [40,41,42]. IFN-γ, which represents a Th1 immune response, is mainly observed in chronic AD and contributes to the perpetuation of skin damage and inflammation [43,44]. IL-17 is a major cytokine related to the Th17 immune response and plays a role in promoting keratinocyte proliferation and amplifying the inflammatory response in inflammatory skin diseases [45,46]. Therefore, in this study, by analyzing the expression patterns of these cytokines, we show that AD is not a disease caused by a single immune pathway but by a complex mechanism, and that CBC is effective in treating AD by regulating this complex mechanism.

Finally, we analyzed the expression and activation of JAK1, JAK2, STAT1, STAT2, STAT3, P-STAT3, and STAT6, which are known to be the major causes of AD to date. The JAK/STAT pathway is a key pathway that mediates various cytokine signals and plays an important role in inflammatory and autoimmune diseases. In particular, the JAK/STAT pathway is essential for regulating various immune responses in AD, including the Th2 immune response [6,8,47]. JAK1 and JAK2 are early stage molecules that transmit signals of various cytokines, such as IL-4, IL-13, IL-6, and IL-33, from receptors to cells, thereby inducing the activation of STAT proteins [48,49,50]. STAT1 and STAT2 are mainly activated in response to IFN-γ and other Th1-related cytokines and are involved in perpetuating skin inflammation and aggravating tissue damage in the chronic stage of AD [51,52]. STAT3 and P-STAT3 are activated by inflammatory cytokines such as IL-6 and IL-23, causing damage to the skin barrier and amplifying the inflammatory response associated with the Th17 immune response. In addition, STAT3 is also involved in the signaling of Th2 cytokines, such as TSLP and IL-33, acting as a central molecule mediating the interaction between Th2 and Th17 immune responses in AD [48,49,52,53,54]. STAT6 plays a critical role in the signaling of IL-4 and IL-13 and regulates key features of the Th2 immune response, such as IgE production, mast cell activation, and eosinophil accumulation. In this study, we systematically analyzed the expression and activation status of JAK/STAT pathway molecules to understand the immunological complexity of AD [6,55,56,57]. In particular, increased expression of activated STAT proteins, such as P-STAT3, indicates hyperactivation of the inflammatory cytokine signaling pathway, suggesting that this is closely related to chronic inflammation and skin damage in AD patients [58,59]. Based on this, it can be suggested that therapeutic strategies targeting specific molecules of the JAK/STAT pathway may be effective in alleviating inflammation and inhibiting disease progression in AD patients.

In conclusion, this study reaffirmed that the JAK/STAT pathway plays an important role in the onset and progression of AD, which was confirmed by analyzing the expression of JAK1, JAK2, STAT1, STAT2, STAT3, P-STAT3, and STAT6. We confirmed that targets related to this pathway were reduced when CBC were topically treated. These results suggest that CBC, which inhibits JAK/STAT, has the potential to be a therapeutic agent for alleviating inflammation in AD.

Although research and regulation of all cannabis-derived ingredients, including CBC, one of the main components of cannabis, is still relatively limited, interest in their therapeutic potential is gradually increasing [60]. In the United States, independent regulations on CBC have not been clearly established, but as research and use of cannabis-derived products with a THC content of 0.3% or less are legalized at the federal level, research on CBC is expected to expand [61]. In the European Union (EU), research and use of cannabis-derived ingredients with THC content not exceeding 0.2% are permitted in cosmetics, and attempts to explore the functionality and potential of CBC are expected to be made within these regulations [62]. In Korea, where research has been conducted, research on cannabis-derived ingredients is also conducted under strict regulations, and research and product development are only possible as long as the THC content does not exceed 0.2%. Although specific regulations and usage guidelines for CBC have not been established, it is likely that they will be dealt with along with regulations for other cannabinoids [63].

Although our study evaluated the therapeutic effects of CBC using a mouse model of DNCB-induced AD, this model is limited in that it does not fully reflect the complex pathological mechanisms of human AD, and the appropriate dose and mechanisms in patients with AD need to be further explored in clinical trials. In addition, although we observed the effects of topical application of CBC at doses of 0.1 mg/kg and 1 mg/kg in this study, these specific doses and modes of administration may also have limitations. Nevertheless, although our study was limited in exploring all the pathways involved in the pathogenesis of AD, it is significant that CBC attenuated the inflammatory response by inhibiting Th2 cytokines (IL-4 and IL-13) and modulating the JAK/STAT pathway, which are currently major therapeutic targets, and could be a potential treatment option to replace or complement conventional steroids or immunosuppressants. Moreover, these anti-inflammatory and immunomodulatory effects suggest that its therapeutic applicability may extend beyond AD to other inflammatory skin diseases.

Finally, given that AD is more common in children, CBC may have greater clinical significance. Unlike THC, CBC is classified as a non-psychoactive substance, making it a potentially safer option for sensitive patient populations, such as children. Although research on CBC is still in its early stages, its strong anti-inflammatory effects suggest its promising medical and industrial applications. As local regulations gradually ease, and awareness of cannabis-derived ingredients continues to evolve, CBC is anticipated to emerge as a viable treatment option for conditions such as AD.

## 4. Materials and Methods

The experimental methods were adapted from previously submitted protocols from the same laboratory, primers, antibodies are provided in the Supplementary Materials [24,64].

### 4.1. Animals

For this investigation, 25 male BALB/C mice were obtained from Japan SLC, Inc. (Shizuoka, Japan). The specimens were six weeks old with a mass range of 16–20 g each. The subjects were divided into five groups, with five mice per group. The animals were maintained in an environment with a 12 h light/dark cycle, at a temperature of 23 °C ± 3 °C and 50% ± 10% humidity. The experimental solution was prepared by combining DNCB (97-00-7, Sigma-Aldrich, Budapest, Hungary) with an acetone/olive oil solution (4:1 ratio) to achieve concentrations of 1% and 0.4%. A 0.03% tacrolimus ointment (Protopic^®^, LEO Pharma, Ballerup, Denmark) served as the positive control. Cannabichromene was provided by Yuhan Care Co., Ltd. (Seoul, Republic of Korea). The Seoul Regional Food and Drug Administration granted permission to use narcotics. Cannabichromene was similarly diluted in the acetone/olive oil mixture to concentrations of 0.1 mg/kg and 1 mg/kg, mirroring the DNCB preparation. To induce AD in the mouse model, the abdomens of the mice were first shaved using animal clippers. Subsequently, 1% DNCB was applied twice over one week. The dorsal hair of the BALB/C mice was then removed using clippers and hair removal cream. The mice were allowed a 72 h rest period before the experiment proceeded. During the experimental phase, 0.4% DNCB was applied to the shaved dorsal skin. After 24 h, treatments of either the vehicle (acetone/olive oil 4:1), Cannabichromene (0.1 mg/kg or 1 mg/kg in acetone/olive oil 4:1), or 0.03% tacrolimus were administered. This process was repeated five times, with a 24 h stabilization period between treatments. Upon conclusion of the experiment, all mice were euthanized. The animal experimental procedures adhered to the Laboratory Animals Welfare Act, the Guide for the Care and Use of Laboratory Animals, and the Guidelines and Policies for Rodent Experiments. These guidelines were provided by the Institutional Animal Care and Use Committee of the School of Medicine, The Catholic University of Korea (approval no. CUMS-2023-0142-01).

### 4.2. Evaluation of Skin Lesions

During the experimental period, the severity of AD-induced lesions was evaluated. The assessment was conducted by three dermatologists who randomly assessed the severity compared to photographs taken immediately after hair removal. The evaluation criteria included (1) the severity of the erythema/hemorrhage, (2) the severity of scarring/dryness, (3) excoriation, and (4) edema. The severity was scored on a four-point scale: 0 (none), 1 (mild), 2 (moderate), and 3 (severe). The final dermatitis score was determined based on the average scores provided by the three dermatologists.

### 4.3. Histological Analysis

Following sacrifice, tissue specimens were immediately preserved in 4% formaldehyde and subsequently embedded in paraffin blocks. These blocks were then sectioned into 4 μm thick slices using a Leica RM 2255 microtome (Wetzlar, Germany). The resulting sections underwent staining with hematoxylin and eosin solution, as well as toluidine blue O solution. A DM2500 LED light microscope (Leica Microsystems, Wetzlar, Germany) was utilized to capture images of the slides. Leica Application Suite X software, https://www.leica-microsystems.com/products/microscope-software/p/leica-las-x-ls/downloads (accessed on 15 December 2024), (Leica Microsystems, Wetzlar, Germany) was employed to measure and assess the epidermal thickness and the quantity of infiltrated cells.

### 4.4. Quantitative Real-Time PCR

Following the euthanasia of the mice, skin samples from their dorsal regions were obtained using a 5 mm biopsy punch (Kai, Seki city, Japan). RNA was subsequently extracted using TRIzol (Invitrogen, Carlsbad, CA, USA). The concentration and purity of the extracted RNA were assessed utilizing a NanoDrop device (Thermo Fisher Scientific, Waltham, MA, USA). Subsequently, the RNA was reverse transcribed to cDNA, which was then combined with target gene primers and Power SYBR^®^ Green PCR Master Mix (Takara Biomedical, Shiga, Japan). Quantitative real-time PCR analysis was performed using a CFX-96 thermocycler (Bio-Rad, Hercules, CA, USA). The PCR protocol comprised an initial 10 min denaturation at 95 °C, followed by 45 cycles of 15 s denaturation at 95 °C and 30 s annealing/elongation at 60 °C. Expression datas analysis was conducted using the cycle threshold method. The expression levels were quantified relative to Actb expression values using the ΔΔCt method based on the cycle threshold [65]. The primer sequences used for quantitative real-time PCR are provided in (Table S1).

### 4.5. Western Blot

Dorsal skin tissue was collected using a 5 mm biopsy punch, similar to RNA collection. The extracted proteins were subjected to BCA assay to measure protein concentration. Then, a constant amount of protein per group was subjected to SDS-PAGE GEL to separate the proteins by size. The separated proteins were transferred to PVDF membranes, and the membranes were blotted with 5% BSA for 1 h at room temperature to prevent non-specific binding. The membranes were then treated with primary and secondary antibodies specific to the proteins. Information about the antibodies is listed in Table S2. The membranes were then treated with secondary antibodies and the bands were visualized using an Amersham™ Imager 600 (GE Healthcare, Chicago, IL, USA). The intensities of all observed bands were calculated using Image J software (version 1.8.0) (U.S. NIH, Bethesda, MD, USA).

### 4.6. Statistical Analysis

Graphical representations were generated utilizing GraphPad Prism 5 (GraphPad Software, La Jolla, CA, USA), and statistical analysis was conducted employing one-way analysis of variance, followed by Tukey’s multiple-comparisons test for subsequent analysis. All data are presented as the mean ± standard error of the mean (SEM). Statistical significance was established at *p* < 0.05 (* *p* < 0.05, ** *p* < 0.01, *** *p* < 0.001).

## 5. Conclusions

**Figure 6:**
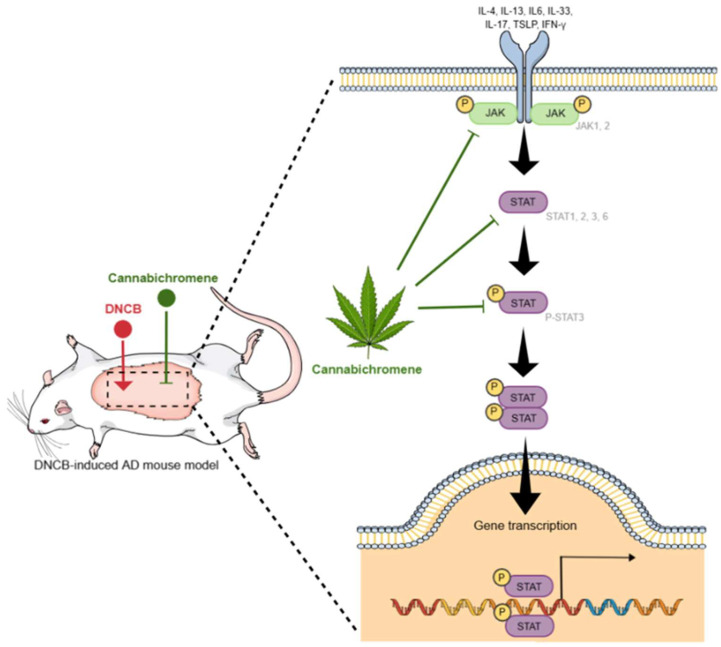
The effect of cannabichromene on a mouse model of hapten-induced contact dermatitis. JAK is activated by cytokine binding to various receptors, and when phosphorylated, STAT protein is activated and phosphorylated. Phosphorylated STAT translocates to the nucleus and activates gene transcription. Cannabichromene has been shown to inhibit these pathways, and, based on these results, it is confirmed to have a therapeutic effect on atopic dermatitis.

In conclusion, our study demonstrated the therapeutic benefits of CBC in a mouse model of DNCB-induced AD. Specifically, CBC was effective in reducing skin severity scores, epidermal thickness, and mast cell counts in an AD mouse model, along with inhibited Th2 cytokines and JAK/STAT signaling (Figure 6). These results suggest that CBC is a potential therapeutic agent for AD.

## Data Availability

The data are included in the article.

## Supplementary Materials

The following supporting information can be downloaded at: https://www.mdpi.com/article/10.3390/ijms252413539/s1.
