# Supplementary material for: Cannabichromene as a Novel Inhibitor of Th2 Cytokine and JAK/STAT Pathway Activation in Atopic Dermatitis Models
Source: Int J Mol Sci. 2024 Dec 18;25(24):13539. doi: 10.3390/ijms252413539 (PMC11677870; doi:10.3390/ijms252413539)
Supplement: Supplementary file 1 [file ijms-25-13539-s001.zip › ijms-3361872-supplementary.pdf]

Table S1. Primer sequences for quantitative real-time PCR amplifications.

Target	Sequence (5'-3')	
Tslp	Forward	AAAGGGGCTAAGTTCGAGCA
	Reverse	AGGGCTTCTCTTGTTCTCCG
Il1b	Forward	TGCCACCTTTTGACAGTGAT
	Reverse	AGTGATACTGCCTGCCTGAA
Il4	Forward	TCTCGAATGTACCAGGAGCCATATC
	Reverse	AGCACCTTGGAAGCCTACAGA
Il6	Forward	CCCCAATTTCCAATGCTCTCC
	Reverse	AGGCATAACGCACTAGGTTT
Il13	Forward	CTGCTACCTCACTGTAGCCT
	Reverse	TATTTCATGGCTGAGGGCTG
Il18	Forward	AGGCATCCAGGACAAATCAG
	Reverse	GGTGTACTCATCGTTGTGGG
Il33	Forward	TCCTGTCTGTATTGAGAAACCT
	Reverse	CTTATGGTGAGGCCAGAACG
Ifng	Forward	TGATTGCGGGGTTGTATCTG
	Reverse	CTGTCTGGCCTGCTGTTAAA
Il17	Forward	ATCCCTCTGTGATCTGGGAA
	Reverse	GCATCTTCTCGACCCTGAAA
Actb	Forward	TGCTAGGAGCCAGAGCAGTA
	Reverse	AGTGTGACGTTGACATCCGT

Table S2. Information on antibodies used in Western blot

Antibody	Dilution	Cat No.	Source
**β-actin**	1:2500	#3700	Cell Signaling Technology®
**JAK1**	1:400	#3344	Cell Signaling Technology®
**JAK2**	1:1000	#3230	Cell Signaling Technology®
**STAT1**	1:1000	#14994	Cell Signaling Technology®
**STAT2**	1:1000	#72604	Cell Signaling Technology®
**STAT3**	1:1000	#9139	Cell Signaling Technology®
**P-STAT3**	1:1000	#9145	Cell Signaling Technology®
**STAT6**	1:1000	#9362S	Cell Signaling Technology®
**Goat Anti-Mouse IgG antibody (HRP)**	1:4000	GRX213111-01	GeneTex
**Goat Anti-Rabbit IgG antibody (HRP)**	1:4000	GRX213111-01	GeneTex
